# Revisiting the Vital Drivers and Mechanisms of β-Glucan Masking in Human Fungal Pathogen, *Candida albicans*

**DOI:** 10.3390/pathogens10080942

**Published:** 2021-07-27

**Authors:** Saif Hameed, Sandeep Hans, Shweta Singh, Ruby Dhiman, Ross Monasky, Ramendra Pati Pandey, Shankar Thangamani, Zeeshan Fatima

**Affiliations:** 1 Amity Institute of Biotechnology, Amity University Haryana, Gurugram 122413, India; saifhameed@yahoo.co.in (S.H.); sandeephans12@gmail.com (S.H.); shweta20.gbu@gmail.com (S.S.); 2 Centre for Drug Design Discovery and Development (C4D), SRM University, Sonepat 131029, India; rubydiman@gmail.com (R.D.); ramendra.pandey@gmail.com (R.P.P.); 3 Department of Pathology and Population Medicine, College of Veterinary Medicine, Midwestern University, 19555 N. 59th Ave., Glendale, AZ 85308, USA; rmonas@midwestern.edu (R.M.); sthangam@purdue.edu (S.T.); 4 Department of Comparative Pathobiology, College of Veterinary Medicine, Purdue University, West Lafayette, IN 47906, USA

**Keywords:** *Candida*, MDR, cell wall, β-glucan, chitin, mannan, PAMP, PRR

## Abstract

Among the several human fungal pathogens, *Candida* genus represents one of the most implicated in the clinical scenario. There exist several distinctive features that govern the establishment of *Candida* infections in addition to their capacity to adapt to multiple stress conditions inside humans which also include evasion of host immune responses. The complex fungal cell wall of the prevalent pathogen, *Candida albicans*, is one of the main targets of antifungal drugs and recognized by host immune cells. The wall consists of tiered arrangement of an outer thin but dense covering of mannan and inner buried layers of β-glucan and chitin. However, the pathogenic fungi adopt strategies to evade immune recognition by masking these molecules. This capacity to camouflage the immunogenic polysaccharide β-glucan from the host is a key virulence factor of *C. albicans*. The present review is an attempt to collate various underlying factors and mechanisms involved in *Candida* β-glucan masking from the available pool of knowledge and provide a comprehensive understanding. This will further improve therapeutic approaches to candidiasis by identifying new antifungal targets that blocks fungal immune evasion.

## 1. Introduction

Human fungal infections, particularly due to *Candida* species, are the foremost source of nosocomial life-threatening infections. Among several *Candida* species, *Candida albicans* is the primary cause of most of these infections [1]. *C. albicans* is a dimorphic, opportunistic fungus that causes candidiasis in immunocompromised patients, particularly among those individuals undergoing chemotherapy, organ transplants, burn injuries, etc. [2]. The current limited antifungal drug regimens include azoles, polyenes, and echinocandins [3]. However, they suffer from the drawback of emerging drug resistance, also known as multidrug resistance (MDR). Under such compelling circumstances of prevailing MDR and where synthesis of new drugs is a lengthy and costly affair, identification of novel antifungal strategies still represents the safer therapeutic option.

The ability of *Candida* to express virulence traits marks the success of the pathogen to cause pathogenicity. These virulence factors include biofilm formation, cell adhesins, hyphal formation, phenotypic switching, exoenzymatic activity (phospholipase, proteinase), and hemolysin production along with immune evasion [4]. To sense and adapt to the hostile niches and evade the immune responses is the basis of infectivity for any fungal pathogen affecting humans, including *C. albicans*. In this context, the cell wall is a necessary component of the cell that not only helps in protection from environmental insults (being the first point of contact) but also against host immune responses. The fungal cell wall is flexible to cope against local environmental inputs offered by the host viz. micronutrient depletion, acidic pH, hypoxia, and alternate carbon sources. This improves the survival of *C. albicans* under a range of variable conditions. This adaptability impacts host–fungus interactions by changing the antigenic determinants that are normally exposed on the cell wall and are recognized by the host immune system, which results in either immune evasion or inflammation. The primary substances reported to cause an immune response are chitin, β-glucans, and mannan, and are uniquely present in pathogenic fungi. In *C. albicans*, the majority of β-glucans are present in the inner cell wall layer which is masked by mannan fibrils located in the outer layer [5]. During β-glucan exposure at the surface, they are recognized by C-type lectin receptor, Dectin-1 [6]; this recognition is a crucial determinant of an antifungal response. To avoid this, the pathogen adopts strategies to evade immune recognition by masking these molecules. This masking of β-glucan polysaccharides from immune detection is a key virulence factor of *C. albicans*.

**Figure 1:**
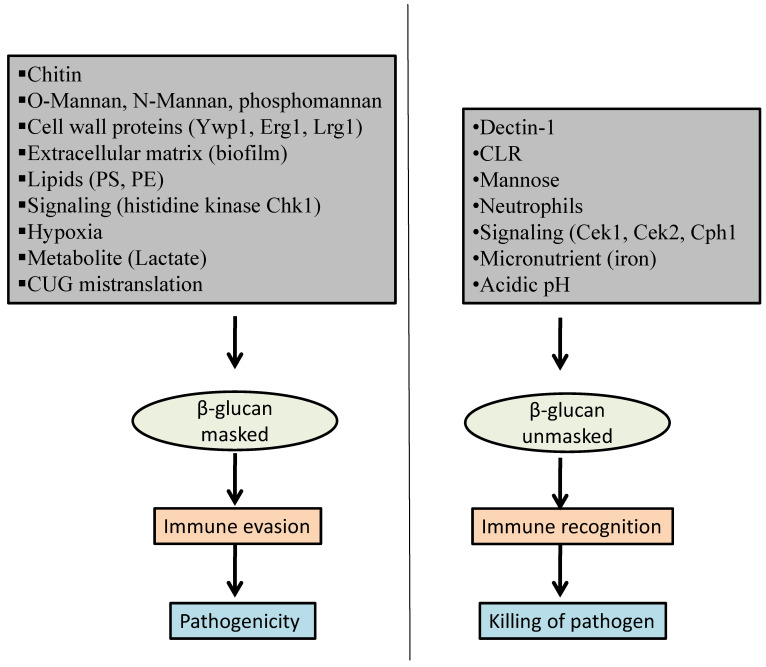
Factors affecting β-glucan masking in *C. albicans*.

A growing appreciation in understanding the biology of host–fungi interaction is emerging. Host immune pathways involved in sensing fungal cell wall components have been characterized for *C. albicans*. This review is an attempt to collate various underlying factors and mechanisms (Figure 1) involved in *Candida* β-glucan masking from an already available pool of knowledge and provides an up-to-date understanding. The following sections will discuss various factors that are known to affect β-glucan masking, thereby presenting a resource to be utilized while designing antifungal therapeutic strategies to block fungal immune evasion.

## 2. *Candida* Cell Wall Components

### 2.1. Glucan

Glucan is one of the most essential parts of the fungi cell surface: it provides mechanical strength to cell wall integrity and represents a composite set-up of polysaccharides, amounting to 50–60% of total dry weight of core structure [7]. *C. albicans* have only β-glucan, which are polymers of glucose units connected by either β-1,3 (65% to 90%) or β-1,6 (10–35%) glycosidic linkages [8]. These polymers of glucan form polysaccharide chains to form a three-dimensional complex. β-1,3 glucan is synthesized at the cell membrane by enzymes known as glucan synthases which are encoded by *FKS1*, *FKS2*, and *FKS3* genes, catalyzing the shift of glucose residues from donor to acceptor molecules. The host immune system has receptors that recognize β-glucan and mount an effective response. Hence, any disturbances in the production and assembly of cell surface architecture lead to glucan layer unmasking, thereby increasing the susceptibility of the fungal pathogen to immune cells [9].

### 2.2. Chitin

Chitin represents 1–2% of the fungal cell wall, consisting of a linear polysaccharide having N-acetyl glucosamine connected by β-1,4 linkages [7,8]. The chitin polysaccharides of fungi form hydrogen bonds that promote stiffening of the carbohydrate into enormously tough fibrous microfibrils. The complex of chitin microfibrils is covalently linked to β-1,3 glucan in most fungal cell walls. Chitin synthesis occurs via the transglycosylation of N-acetylglucosamine residues from substrate UDP-N-acetylglucosamine into a polysaccharide chain [10]. The enzymes involved are chitin synthases encoded by *CHS1*, *CHS2*, *CHS3*, and *CHS8* in *C. albicans*, which deposits the newly synthesized chitin in the extracellular space near the cell membrane.

### 2.3. Mannan

Mannan forms the external covering of the fungal cell surface and is less rigid than β-glucans and chitin, hence having a limited effect on cell shape. However, its low porosity and permeability does affect the entry of antifungal drugs and defense mechanisms [11]. Additionally, being the outer most layer, it is assumed to be significant in β-glucans masking and evading host immune detection [12]. Mannose is integrated into three structures: linear O-linked mannan, extremely branched N-linked mannan, and phospholipomannan. Initially, mannose is added to a dolichol phosphate acceptor, followed by incorporation into N-, O-mannosylation, and GPI (glycosyl phosphatidylinositol proteins) anchors. N-linked mannans are assembled from α-1,6-mannose backbone with α-1,2-oligomannose sidechains capped with β-1,2- mono-, di-, tri-, or tetra mannans [13]. Mannose residues are added to serine or threonine residues to produce O-linked mannans. Phospholipomannans do not contain glucosamine and show associations with glycan chains.

## 3. Factors That Influence β-Glucan Masking

### 3.1. Host-Pathogen Interaction

#### 3.1.1. Dectin-1

Any invading pathogen, like *Candida*, faces a significant challenge from the host immune system. Pathogen recognition receptors (PRR) identify particular fungal pathogen-associated molecular patterns (PAMP), which include essential and specific fungal cell wall components such as glucan, chitin, and mannan (PRR). When fungi infect our bodies, the cell wall is crucial in triggering an immune response. The inner cell wall of fungi is high in glucan, which is responsible for triggering an inflammatory response, but immune cells are unable to recognize it. As soon as the -glucan is exposed, it is recognized by Dectin-1, a C-type lectin PRR [14,15]. When Dectin-1 recognizes β-glucan, myeloid cell signaling is activated along with the phagocytic response, and a pro-inflammatory cytokine response is introduced. Other activities for destroying fungal cells by neutrophils and macrophages are also initiated via their reactive oxygen (RO) and reactive nitrogen (RN) species [16,17]. Dectin-1 receptor shows an association with β-glucan, whereas the Dectin-2 receptor is associated with the α-mannan, which is found on the fungal cell’s outer covering [14,16]. With the aid of macrophages, dendritic cells, and neutrophils, Dectin-1 receptors boost innate immunity against *Candida albicans* [17,18]. Macrophages are essential in the detection of invading fungal cells. Si Min Chen et al. found that macrophages lacking Dectin-1 were unable to mount an effective response to *C. albicans* stimulation [19]. Ywp1 is one such component that helps the glucan particle remain masked and guards against immune cells’ identification [20,21]. In deprivation of O-mannan from the fungal cell wall, β-glucan gets unmasked and eventually gets recognized by Dectin-1, resulting in expanded phagosome maturation [22]. Nina Klippel and team showed that Chk1p is responsible for the masking of mutant β-glucan to prevent recognition by the immune cells [23]. Dectin-1 triggers CR 3 and S1GN-R1 to generate signaling. Dectin-2 and -3, on the other hand, have a synergistic effect on inflammatory responses [24]. Dectin-1 binds soluble β-glucan and particulate β-glucan, a ligand that is similar to β-glucan found in cell walls. In response to glucan-coated beads, dendritic cells (DCs) release reactive oxygen species (ROS). This response can be produced by glucan particles with a diameter of 500 nm or larger, but not by particles with a diameter of 200 nm or smaller. It indicates that glucan detection can be dependent on the physical properties of glucan at nanometric scales. However, the precise dimensions of β-glucan exposure necessary for Dectin-1 activity are still unknown. Since Dectin-1 signaling is ligand size-dependent, masked β-glucan exposures may be limited to non-stimulatory sizes, while unmasking processes may result in larger β-glucan exposures that are more effective at activating Dectin-1. This highly immunogenic molecular pattern is linked to dendritic cell, macrophage, and neutrophil activation, primarily by Dectin-1 and also by β2-integrin [17]. PRRs help DCs and other leukocytes identify the polysaccharides found on fungal cell walls. Dectin-1 and the DCSIGN transmembrane C-type lectins (CTLs) are the proteins bound to β-glucan and mannan, respectively. Dectin-1 detects β-glucan, triggering an immunogenic response that leads to cellular activation in innate immune cells and fungal phagocytosis. When PRRs are activated, DCs enter the picture and take care of both innate and adaptive immunity by guarding pathogen entry. As a result, controlling the amount and exposure of immunogenic cell-wall ligand is an important feature of the host-pathogen relationship that requires further investigation.

#### 3.1.2. C-Type Lectin Receptor (CLR)

Infected patients are known to have inadequate C-type lectin receptors (CLRs) activity, and the CLRs are crucial in achieving antifungal immunity [25]. CARD9 is an intracellular signaling pathway for CLRs that plays an important role in preventing fungal infections [26]. When TLRs and CLRs are co-stimulated, the MyD88 and Syk/CARD9 pathways are activated, which improves the inflammatory response [24]. When these PAMPs are identified, the innate immune system is stimulated, involving phagocytic cells and the production of adaptive immunity at the same time [27]. A fungal invasion and an imbalance in the host defense response cause systemic candidiasis. CLRs, dendritic cell-specific intercellular adhesion molecule 3-grabbing non-integrin (DC-SIGN), Mincle and Galectin-3, mannose receptor (MR), complement receptor 3 (CR3), and Toll-like receptors (TLRs) are all PRRs that are responsible for the host immune system recognizing the fungal species. Mutations in the CLR pathway are linked to invasive fungal infections among these PRRs.

#### 3.1.3. Mannose Receptor

This receptor can recognize mannose structure and is found on the surface of macrophages. The action of macrophages is affected by the exposure of glucan present on the fungal surface. The structure of mannan molecules plays a major role in glucan exposure. If mannan has simple molecular structure, it enhances the chances of exposure [5]. This receptor detaches from the surface of macrophages and is released into the environment to help our immune cells recognize fungi. Mannan behaves as an immune guard for macrophages that installs Dectin-1 as the primary immune recognition receptor [28]. Interleukin 6, 10, and INF-α, and TNF-γ are all produced by TLR 4 in conjunction with the mannose receptor. Dectin-2, -3 also acts as a dimer in RAW264.7 macrophages to identify mannans and secrete INF-α [29]. The primary unit found in N-mannose for immune recognition and fungus virulence is α -1, 2- mannose [30]. The stimulation of T-helper 1 (Th1) cytokine synthesis is thought to be mediated by mannose receptors. Macrophages ingest and kill *C. albicans* thanks to mannose receptors involved in TNF-α, IL-1, IL-6, and granulocyte-macrophage colony-stimulating factor (GM-CSF) responses. Human keratinocytes also express a mannose-binding receptor that causes anti-*Candida* activity, most likely through the development of nitrous oxide. During in vivo mouse studies of candidiasis, these receptors play a role in infection. In mannose-receptor-knockout mice, infection with *Candida albicans* via intraperitoneal injection leads to an increase in fungal burden [31].

#### 3.1.4. Neutrophil Extracellular Trap (NET)

One aspect of the mechanisms required for altering epitope unmasking involves neutrophils causing changes in fungi’s innate pattern recognition. It sheds light on how the *C. albicans* cell wall reacts to the threat of a host immune response during disseminated candidiasis. This also leads to useful knowledge on host-pathogen responses during systemic infection. In a mode of neutrophil attack against *C. albicans* and other fungi in vivo and in vitro, neutrophils form neutrophil extracellular traps (NET) comprised of DNA and other antimicrobial components. While mouse neutrophils produce less NET than human neutrophils, neutrophil proteases are a key element of NET production in certain circumstances, and neutrophil elastase trafficking is controlled during NETosis (release and activation of neutrophil extracellular traps) toward *Candida albicans*. However, neutrophils from dipeptidyl peptidase (DPPI)-deficient mice—which are necessary for the activation of the three major neutrophil proteases: elastase, cathepsin G, and proteinase—showed no difference in their capacity to trigger β-glucan unmasking, chitin deposition, or streptavidin deposition. Thus, in this mechanism, these three proteases play a key part in the downstream cell wall remodelling caused by neutrophil assault. The aspartic proteases formed by *C. albicans* are chemotactic agents for neutrophils and are likely involved in their modulation through ROS generation. Furthermore, major components of the *C. albicans* cell wall can play a role in NETosis pathway activation. β-glucans, which are recognized by neutrophil Dectin-1 receptor, induce NET release, most likely through an ROS-independent mechanism [32]. However, the precise roles of each type of surface or secreted *C. albicans* components in NET formation are still unknown. Dectin-1 binding enhancement induced by neutrophils can alter the secondary immune response to *Candida albicans,* or the binding may not affect the response due to unnecessary recognition modalities. *C. albicans* use epitope masking and have developed innovative ways to escape pattern recognition receptors by covering unique epitopes. Several groups have identified how the fungal cell wall architecture restricts detection of the β-glucan sugar by immune receptors such as Dectin-1, a C-type lectin critical for fungal infection resistance [33]. It was discovered that patients with active symptoms had higher availability of -glucan, as well as an accumulation of neutrophils at the site. According to new research, neutrophils increase -glucan exposure in *C. albicans*, resulting in a stronger immune response. This means that during infections, the *C. albicans* cell wall will change due to environmental factors, which cause the fungal cell wall’s alterations, making it easily detectable for the Dectin-1 receptor [34], and the neutrophils are triggered when the fungal cell wall changes. Neutrophils begin the destruction of cell walls, assisting macrophages and cytokines in the destruction of fungal cells [33].

It has been demonstrated that neutrophils can detect fungi invasion early on and initiate an immune response. The phagocytosis of the fungal pathogen by innate immune cells is critical for the effective clearance of *C. albicans* from the host tissue. Pathogen identification triggers a multi-step mechanism called phagocytosis. This results in pathogen engulfment, phagosome maturation to remove the pathogen, and then phagolysosome resolution, which has been thoroughly reviewed by Levin et al. [35]. They use the MEK/ERK cascade to recognize invasive filamentous *C. albicans*, which causes macro type-specific neutrophil activation [36]. In comparison to the non-filamentous mutant fungus, these filamentous *C. albicans* types can manipulate phagosomal maturation [37]. Another study found that anoxic conditions reduced specific polymorphonuclear neutrophil (PMN) functions directed against *C. albicans*, such as NET formation, ROS triggering, phagocytosis, and the ability of *C. albicans* to form biofilms; however, as previously demonstrated for biofilms formed under normoxic conditions, anoxically formed biofilms can inhibit PMN functions. Infections in anoxic conditions confirmed the existence of ROS-independent pathways of NET induction. Fungal MAP kinase-driven responses, especially via Hog1, are triggered by NET-induced damage, limiting cell wall remodelling machinery. It has been discovered that -glucan unmasking necessitates NET moderated interference, which activates a change in the fungal cell wall that makes it easily identifiable for the Dectin-1 receptor [33]. NETs are more harmful to hyphae forms than they are to yeast forms. In the early stages of interaction, *C. albicans* stimulate NETs through autophagy and ROS [38,39]. Neutrophils have thus been established as one of the important factors for alterations in the fungal cell wall in a variety of studies.

### 3.2. Cell Wall Proteins

The β-glucan masking involves the role of some cell wall proteins under certain conditions. Ywp1 (yeast wall protein) is present in abundance in the cell wall of the yeast form of *Candida,* involved in β-glucan masking from immune recognition as it remains intact in the cell wall. Thus, there is less exposure of β-1,3-glucan by host antibodies, which favors more masking. Experimental studies showed that when antibody binding to β-1,3-glucan was more in germ tubes, very little or no Ywp1 was present, resulting in more β- glucan unmasking than in yeast forms. Ywp1 has an antiadhesive effect along with a masking effect [21]. Another protein, endo-1,3-β-glucanase Eng1, is also involved in β-glucan masking and studies demonstrated that in the absence of this protein, there is enhanced recognition byDectin-1 in host cells [40]. Lrg1is a protein encoding a GTPase-activating protein (GAP) which downregulates the GTPase Cdc42 and downstream MAPKKK and Ste11, representing a regulator of the Cek1 pathway. The study showed that hyperactivation of Cdc42in lrg1 mutant results in more exposure of β-glucan [41]. It has been demonstrated that deletion of this *LRG1* gene leads to disruption of the Cek1 pathway and β-glucan becomes unmasked to become more prone for immune recognition by host cells. Lrg1 isa repressor of β-glucan unmasking which can in turn induce elevated levels of TNF-α secretion from murine macrophages. Rho1 is a regulatory subunit of the β-glucan synthase enzyme that directly controls biosynthesis of the cell wall through activation of catalytic subunits like Fks1 [42].

Kex2is a serine protease enzyme which resides in Golgi and is Ca^2+^-dependent and required for the proprotein dispensation step during maturation of protein prior to exiting the trans-Golgi network and does cycling through Golgi vesicles and late endosomal compartment [43]. It has been demonstrated that loss of the gene *KEX2* leads to changes in organization of cell structure and negatively affects the N-linked mannosylation pathway. A Kex2∆ null mutant showed the amplified levels of β-glucan and thus partly exposes the surface of the cell, which accounts for the augmented IL-10 production in the interaction of cell and macrophage [44,45].

### 3.3. Chitin

Fungi can alter the broken structure of a cell by an alteration in chitin content to sustain cell wall integrity. In *C. albicans*, the immune recognition via mononuclear cells is blocked by chitin [46]. The unmasking of β-glucan leads to enlarged exposure of chitin content on the surface of *C. albicans.* Consequently, the antifungal drug echinocandin’s mechanism of resistance is by increased chitin biosynthesis [47]. It has been previously reported that caspofungin susceptibility is reduced with elevated chitin content in the cell surface of *C. albicans* in a mouse model of systemic infection. Additionally, the susceptibility of echinocandins is reduced with increased in-cell-wall chitin content of *C. albicans*, both in vivo and invitro [47]. The cell-wall-specific antifungal that targets the biosynthetic pathway disturbs the cell structure through β-glucan exposure, unmasking, or cell wall disorganization [48]. Caspofungin reduced the biosynthesis of β-glucan, which resulted in reduced levels of β-glucan in the cell [47]. This disturbance in the cell structure mechanism can cause more exposure of β-glucan. Even caspofungin-targeted cells elicited the TNF-alpha generation, which suggested that β-glucan unmasking by antifungal drug caspofungin induces the proinflammatory response. Hence, caspofungin may hit at *Candida* in a double way, where on one hand, it kills them at high concentrations but in lower concentrations leads to unmasking of β-glucan.

### 3.4. O-Mannan, N-Mannan, and Phosphomannan

In the architecture of the *C. albicans* cell wall, the outermost layer is composed of proteins that are covalently attached with O-linked or N-linked mannans, also known as mannoproteins. O-linked mannans are short, linear oligosaccharides which are an arrangement of seven α-1,2-mannose units. N-linked mannans are well-branched oligosaccharides incorporated with residues of mannose with several glycosidic linkages [49]. The importance of these N-linked and O-linked mannans’ structures lies in their involvement in the immunological distinctiveness of *C. albicans* and is understood as molecular patterns that interrelate with PRRs of the innate immune cells. They are immunostimulatory and involved in the identification and uptake of fungal cells by the immune cells and system. It has been reported that O-linked mannans are recognized by Toll-like receptor TLR4, whereas N-linked mannans are recognized with mannose receptors, Dectin-2, and Dectin-3, DC-SIGN [50,51]. Apart from sensing of β-glucan, it has been reported that macrophages also require the recognition of phosphomannan that negatively affects the phagocytic process. A study also reported that O-linked mannans detrimentally impact macrophage engulfment of *C. albicans* [44]. Phosphomannans are the β-1,2-mannose moiety which are bonded to branching N-glycan through a phosphodiester bond. They are recognized by immune cells and stimulate cytokine secretion [30]. A study [22] demonstrated a mutant in which there is loss of cell wall O-mannan, which results in enhanced attainment of phagosome maturation markers and weakens the hyphal growth within macrophage phagosomes. This brings macrophage change in actin dynamics and thus makes fungal cells incapable of escaping macrophages.

### 3.5. Extracellular Matrix

The *Candida* biofilms possess an extracellular matrix that is assembled from a polysaccharide complex which cooperates in the assemblage and maintenance of the matrix. In *C. albicans*, the polysaccharides present in the cell wall are β-glucan and mannans. It was mentioned that glucan is the major contributor for drug resistance in biofilm-mediated drug resistance [52]. The matrix β-1,6-glucans were linear in the biofilm matrix, in contrast with the branching structures located inside the cell wall. The role of β-1,3-glucan is vital in biofilm-mediated defense of *C. albicans* against host immunity and antifungal treatment [53].

### 3.6. Lipids

Lipids are an important component of membrane phospholipids in the cell membrane of *Candida*, so it might be interpreted that any disturbances in lipid homeostasis might affect the lipids or their related pathways, which can affect masking of β-glucan. It is known that PS (Phosphatidylserine) and PE (Phosphatidylethanolamine) make up 6% and 15% of total membrane phospholipids, respectively [54]. It is known that PS modulates certain signaling pathways which regulate the cell wall, such as the calcineurin signaling pathway, protein kinase C pathway, Hog1p, or Cek1p pathway [55]. A study demonstrated that a post-transcriptional regulator called Ccr4-Pop2 mRNA deadenylase is required for maintenance of cell wall integrity. The mutants of this regulator showed that defects in the functioning of Ccr4-Pop2 also relate to defects in phospholipid homeostasis and dysfunctional mitochondria. Additionally, Ccr4-Pop2 also plays a role in cell wall biogenesis, which correlates with the β-glucan activity and interlinks with mitochondria and phospholipid homeostasis [56]. A study has reported that PS is necessary for β-glucan masking against host immune system recognition. PS is encoded by PS synthase gene called *Cho1*. The loss of function of PS leads to decreased β-glucan masking and helps in enhancing immune recognition by host cells. There is also an increase in unmaskedβ-glucan exposure on the cell surface, evident from increased binding to Dectin-1 receptor and increased secretion of TNF-α by macrophages [57]. The PS synthase enzyme (Cho1) and PE are both crucial in the pathogenicity of *C. albicans.* Additionally, mutant CHO1 showed higher β-glucan exhibitions than non-mutant. This permits recognition to a greater extent by Dectin-1 and as a result, produces elevated pro-inflammatory responses [18].

### 3.7. Signaling Pathways

Several signaling pathways are also involved in host pathogen interaction and immune recognition by host cells. The MAPK pathways which include the cascade of Ste11-Hst7-Cek, Cek1 MAPK, have been reported to regulate β-glucan masking [58]. Two components of MAPK pathways such as Cek1 and Cek2 play vital roles in masking of β-glucan through disorganization of the external mannoprotein layer [59,60]. However, on the other hand, the hyperactivation of Cek1 can lead to unmasking of β-glucan. A transcription factor Cph1 encoded by *CPH1* gene negatively regulates β-glucan masking and may cause unmasking upon hyperactivation [18]. A two-component signaling pathway called histidine kinase contributes to biosynthesis of the cell wall and virulence of *C. albicans*. In this pathway, several kinases are involved, such as CaSln1p, Cos1p, and Chk1p. They are also associated with the regulation of mannan biosynthesis [61,62]. However, Chk1pencoded by gene *CHK1* is crucial for activation of phagocytes and represents an attractive target as there is no human homologue. A study by Klippel et al. also demonstrated that Chk1p is not only involved in maintaining the escape of *C. albicans* from ingestion by immune cells, but it also participates in blocking another event, such as phagosome maturation. The histidine kinase Chk1p plays a crucial role for masking of β-glucans in *C. albicans* from phagocyte recognition [23].

### 3.8. Micronutrient

During infection, *C. albicans* encounters various anatomical niches which are micronutrient depleted. One such factor is the availability of iron, as it is not freely available due to its transitional nature. It has been studied that the recognition of the fungal cell wall and stimulation of the cytokine by host immune cells is regulated by the iron, and iron-depleted conditions can induce β-glucan masking [63,64,65]. High iron-containing media showed higher levels of exposed β-glucan in comparison to a limited iron condition. The glucose media with a limited iron condition exhibits decreases in mitochondria activity due to the presence of various heme iron-containing proteins [66]. This leads to changes in cellular metabolism towards fermentation mode, which increases lactate production, while in cases of high iron-containing media, a reduction in lactate production occurs [67]. It is suggested that glucose-grown, high iron-containing cells have improved mitochondrial activity, which can reduce lactate-mediated β-1,3-glucan masking. Iron-rich media also reduce the level of total mannans as well as phosphomannans in the outer cell surface, thereby increasing β-1,3-glucan. *Gpr1* and *Crz1* genes are mediated by exogenous lactate and target β-1,3-glucan masking [68]. The change governed by iron that induces β-glucan exposure is affected by intracellular L-lactate. This change is GPR1-independent, but Crz1-dependent [69]. β-glucan masking can be affected by varying zinc concentrations, where zinc deprivation increases β-glucan exposure.

### 3.9. Hypoxia

Hypoxia (low oxygen) is another stress condition that infectious fungi face inside the host [70]. *C. albicans* shows a high resistance to hypoxia [16], and accordingly, these infectious fungi can inhabit a hypoxic environment such as the gastrointestinal region [71], in addition to aerobic environments including the skin and mucosa. Hypoxia conditions lead to improvements in virulence and β-glucan masking on the fungal cell surface. Additionally, Efg1 plays an important role in the regulation of the hypoxic stress in *C. albicans*, [72] and Hog1 pathway has a role in control of the hypoxic responses in *Saccharomyces cerevisiae* and human cells [73]. β-glucan masking is induced by hypoxia and is dependent and regulated by cAMP- protein kinase A signaling pathway. cAMP plays a crucial function in cell surface integrity, yeast to hyphae transformation, and stress adaption [74]. It is suggested that under hypoxic conditions, *C. albicans* enhances its survival by avoiding polymorphonuclear leukocyte attacks through β-glucan masking [75]

It has been reported that more polymorphonuclear leukocytes at the site of an infection promote less oxygen, which turned out to be beneficial for the fungi as phagocytic cells could not produce ROS [75]. Low oxygen conditions improve *C. albicans’* survival due to better masking of β-glucan and avoidance of Dectin-1 recognition. As a result, less phagocytosis by macrophages occurs and there is a drop in production of IL-10 and TNF-α. This suggested that *C. albicans* and host interactions are remarkably affected by β-glucan masking that is promoted by hypoxia. Generally, β-glucan masking depends on cAMP-PKA, and the mitochondrial signaling pathway which regulates local immune response and supports fungal assembly [76].

### 3.10. pH

*C. albicans* colonizes niches like the abdomen, vagina, and gastrointestinal traits, which have varied pH *C. albicans* tends to show that growth within the acid atmosphere involves renovation of plasma membranes that manufacture exaggerated chitin and β-glucan on the cell wall. Vaginal mucosa has a pH ranging between 4–5, depending on age and origin [77]. A study on the pH alteration on *C. albicans* explored that exposure to acidic conditions induces the contact of β glucan and chitin, which are usually hidden below the mannan fibrils [78]. Both factors like β-glucan and chitin are recognized by the natural immune response [68] and at low pH, *C. albicans* activates a strong pro-inflammatory natural immune reaction and improved neutrophil recruitment [79]. At present, the defined cell wall remodeling because of acidic pH on cell surface unmasking is unidentified. Visualization of the configuration of *C. albicans* revealed that the mannan fiber was condensed due to acidic conditions, which may be the reason for less mannan production. Rim 101 is a signal transduction pathway known to be involved in cell wall remodeling by proper localization of chitin in the cell wall apart from its usual role in governing pH response. Due to the involvement of Bcr1-Rim101-dependent signal cascade in chitin localization through regulation of chitinase Cht2p, it is hypothesized that this transcription factor may also be involved in β-glucan unmasking in a pH-dependent manner [78].

### 3.11. Metabolites

The structural design of the *C. albicans* cell surface gets altered extensively due to carbon sources. For instance, cells grown on lactate acquire thinner, smaller amounts of flexibility in cell walls and an altered immune response compared to complex media such as glucose-grown cells [80,81]. The ratio of the β-glucan compared to chitin and mannan remains similar [80], as exposure of β-glucan on the cell surface might be affected by the carbon source. Further analysis exposed that *C. albicans* cells activate PAMP β-glucan masking at the cell wall in response to functional levels of lactate, produced either by host cells or fungal cells [82]. The β-glucan masking response induced by lactate is linked with an attenuation of inflammatory reaction and neutrophil recruitment. This masking, which is induced by lactate, is regulated by a signaling pathway that plays an important role in mechanisms other than well-established signaling pathways [82]. A recent study demonstrated that lactate-induced masking of β-glucan is governed by exoglucanase enzyme Xog1p that digests β-glucan epitopes to enhance immune evasion, thereby representing an antifungal target [14]. Metabolism of lactate is crucial for β-glucan masking and its stimulation depends on uptake using glyoxylate cycle enzyme isocitrate lyase [81] and Jen1/2 transporters [83]. Gpr1p receptor helps in the recognition of lactate, which is present in the cyclic AMP-protein kinase A pathway that activates lactate-induced β-glucan masking and virulence factor [84]. Consequently, *Cag1* and *Gpa2* genes may alter the function of Gpr1 signaling for masking of β-glucan and cyclic-AMP pathways through a process that remains to be defined. Lactate signaling is also influenced by involvement of the factor Crz1p, which controls cell surface integrity, drug tolerance, cell wall remodeling, and the specific response to calcium ions through calcineurin signaling [85]. The decreased wall β-glucan levels being less vulnerable to caspofungin signifies that environmental variation to different sources of carbon may control therapeutic options.

### 3.12. Mistranslation of CUG

The interaction of the host with the pathogen is also influenced by CUG mistranslation. In *C. albicans*, mistranslation of CUG occurs due to dual coding for serine (97%) and leucine (3%). The CUG mistranslation helps in masking the β-glucan molecule present in the cell wall, which is generally detected by the host immune system [6,86]. The studies showed that manipulation of genetic ambiguity results in increased expression of virulence factors such as morphogenesis, phenotypic switching, and cell adhesion [87]. The increase of leucine, a hydrophobic amino acid, results in the increased adherence of the pathogen to the host cell. The changes brought by CUG mistranslation influences the exposure of β-glucan on the surface of cells and reduces macrophage phagocytosis of *C. albicans*. It has been reported that the rise in adherence is interceded by two mechanisms. Firstly, CUG mistranslation increases cell surface hydrophobicity, which is crucial in the attachment of *C. albicans* to abiotic surfaces and host cells [88,89]. Secondly, CUG mistranslation enhances the substrate strength of adhesins like Als proteins. The presence of Als3p-Leu in yeast promotes more adherence in comparison with the expression of Als3p-Ser. CUG mistranslation enhances diversity of structures and modulates the adherence of other cell wall proteins. The functional variability of CUG mistranslation may widen the array of host substrates, of which *C. albicans* can bind through, manipulating the interaction of the ligand region. Previous studies reported that the extent of leucine misincorporation at CUG codons fluctuates, and greatly depends upon external conditions such as temperature and pH [87]. CUG mistranslation confers an advantage by increasing the capability of cells for adhering to a wide range of host substrates and minimizing connections with immune effector cells. This factor has not only provided greater diversity in proteins on the cell surface of *C. albicans* but also contributes to increasing the range of metabolic responses of *C. albicans*. Thus, CUG mistranslation expands the host micro niches in which *C. albicans* can thrive well to survive [90].

## 4. Conclusions

Given the prevalent MDR scenario in *Candida* spp., boosting of the immune response by exposing factors manipulating the β-glucan masking phenomenon endorsed in this review presents an alternative approach that needs attention (Table 1). A deeper insight of the details of cell wall structure and β-glucan masking is likely to indicate new potential therapeutic targets. Furthermore, understanding the mechanisms and effects of the host immune evasion strategies used by *Candida* spp. could indicate new ways in which β-glucan masking could be blocked. Understanding these mechanisms could provide a promising means of augmenting antifungal immunotherapies. Therapies to enhance β-glucan exposure will aid in controlling *Candida* infections. These could potentially include, for example, specific fungal polysaccharides that provide immuno-amelioration for fungal diseases. The influence of cell wall components in the fungi-host relationship and their part as a target for the next generation of antifungal drugs may provide a platform for future research and pave the way for better management and improvement in therapeutic strategies. Together, the cumulative mechanisms underlying β-glucan remodeling will help manipulation pharmacologically to influence immune recognition and infection outcomes.

## Figures and Tables

**Table 1:** Key players of β-glucan masking mechanisms in *C. albicans*.

Factors Affecting β-Glucan Masking	Key Players	References
Dectin-1	Myeloid cell signaling, phagocytosis, proinflammatory cytokine release	[16,17]
CLR	CARD9 signaling	[26]
Mannose	Dectin-2, -3, macrophages	[29]
Neutrophils	NET, phagocytosis	[32,33]
Cell wall protein	Ywp1p, Eng1p, Lrg1p, Kex2	[21,40,41,44,45]
Chitin	Mononuclear cells, caspofungin	[46,47]
O-mannan, N-mannan and Phosphomannan	TLR4, Dectin-2, Dectin-3, DC-SIGN, phagocytosis	[50,51]
Extracellular matrix	Biofilms	[53]
Lipids	Ccr4-Pop2, PS, PE	[56,57]
Signalling pathway	Cek1, Cek2, Cph1, Chk1	[18,23,59,60]
Iron	Crz1	[63,64,65]
Hypoxia	Efg1	[72]
Acidic pH	Neutrophil, Rim101	[78,79]
Metabolite	Lactate	[82]
CUG mistranslation	Leucine	[88,89]

## Data Availability

Not applicable.
